# Synergistic Potential of Contamination Remediation and Carbon Fixation: Functional Resilience of Carbon Fixation in Petroleum Hydrocarbon-Degrading Microbial Communities Under Enhanced Natural Attenuation

**DOI:** 10.3390/microorganisms13092205

**Published:** 2025-09-20

**Authors:** Pingping Cai, Shuang Gan, Zhuo Ning, Min Zhang

**Affiliations:** 1 School of Water Resources and Environment, Hebei GEO University, Shijiazhuang 050031, China; cppyjy@163.com; 2 Hebei Province Collaborative Innovation Center for Sustainable Utilization of Water Resources and Optimization of Industrial Structure, Shijiazhuang 050031, China; 3 Hebei Province Key Laboratory of Sustained Utilization and Development of Water Recourse, Shijiazhuang 050031, China; 4 Institute of Hydrogeology and Environmental Geology, Chinese Academy of Geological Sciences, Shijiazhuang 050061, China; ganshuang2016@163.com (S.G.); ningzhuozhuo@163.com (Z.N.); 5 Key Laboratory of Groundwater Remediation of Hebei Province & China Geological Survey, Shijiazhuang 050061, China

**Keywords:** enhanced natural attenuation (ENA), carbon-fixing microorganisms, petroleum hydrocarbon, microbial community, functional gene

## Abstract

Enhanced Natural Attenuation (ENA) can accelerate pollutant degradation by adding electron acceptors or nutrients. However, its impact on carbon-fixing microorganisms, which are widely found in the natural attenuation process, remains unclear. In this study, four types of ENA materials were added in batch experiments. Chemical analysis and metagenomic sequencing were employed to analyze the degradation kinetics of petroleum hydrocarbons, the consumption pattern of nitrate, as well as the functional genes and population evolution characteristics of carbon-fixing microorganisms. Results showed that nitrate-based enhancement materials significantly improved the petroleum hydrocarbon degradation rate but suppressed the expression of some carbon fixation genes, such as those involved in the Calvin–Benson–Bassham cycle. Nevertheless, the overall abundance of carbon fixation genes did not show a notable decline. Dominant bacterial genera such as *Pseudomonas* and *Achromobacter* possessed both hydrocarbon degradation and carbon fixation capabilities. Although the calcium peroxide treatment group only achieved a 40% petroleum hydrocarbon degradation rate, it significantly promoted the abundance of carbon fixation genes involved in the reductive tricarboxylic acid cycle pathway. Therefore, ENA alters carbon fixation pathways but does not diminish carbon fixation potential, indicating its potential for synergistically achieving pollution remediation and carbon fixation.

## 1. Introduction

The migration and diffusion of petroleum hydrocarbon contaminants in aquifers pose a persistent threat to groundwater resources [1]. Natural attenuation (NA), as a cost-effective remediation strategy, relies on microbial metabolism to achieve pollutant degradation. Recent studies have revealed the presence of a substantial number of CO_2_-fixing microorganisms in petroleum-contaminated aquifers. These microorganisms fix inorganic carbon through pathways such as the Calvin cycle and the reductive acetyl-CoA pathway, forming potential biological carbon sinks [2,3,4,5]. For instance, sulfur-oxidizing bacteria, nitrifying bacteria, and certain iron-reducing bacteria can simultaneously drive hydrocarbon degradation and carbon fixation [6,7,8], suggesting that the natural attenuation process may possess dual functions of pollution remediation and carbon sequestration.

However, the remediation period of traditional natural attenuation often spans several decades, making it difficult to meet the urgent management needs of contaminated sites [9]. To accelerate the remediation process, enhanced natural attenuation (ENA) is achieved by adding electron acceptors (such as O_2_, NO_3_^−^, SO_4_^2−^) or nutrients (N/P sources) to stimulate the metabolic activity of indigenous microorganisms [10,11]. Numerous empirical studies have demonstrated that nitrate enhancement can significantly increase the degradation rates of benzene, toluene, ethylbenzene, and xylenes (BTEX) and alkanes, leading to a notable increase in the abundance of hydrocarbon-degrading bacteria [12]. Nevertheless, current ENA research has predominantly focused on pollutant removal efficiency, neglecting the impact on key carbon-fixing functional microorganisms.

There are significant biochemical pathway interactions and conflicts between carbon-fixing microorganisms and hydrocarbon-degrading microorganisms. On one hand, most carbon-fixing bacteria (such as *chemolithoautotrophic Nitrospira* and *Thiobacilli*) utilize O_2_, NO_3_^−^, or Fe (III) as electron acceptors and H_2_, H_2_S, or Fe^2+^ as electron donors, which highly overlap with the nitrate-based materials commonly used in ENA [4,13,14]. Theoretically, the addition of electron acceptors may simultaneously activate carbon-fixing pathways and promote the formation of biological carbon sinks [15,16,17]. On the other hand, heterotrophic hydrocarbon-degrading bacteria (such as *Pseudomonas* and *Acinetobacter*) proliferate rapidly under nutrient-rich conditions, potentially inhibiting carbon-fixing microorganisms through mechanisms such as competing for electron acceptors (e.g., NO_3_^−^) and ecological niches, secreting antibiotic-like secondary metabolites to alter community structure, or rapidly consuming electron acceptors to cause local redox potential changes. This conflicting interaction makes the succession direction of carbon-fixing microbial communities during the ENA process unclear [18,19,20].

Therefore, this study selected sandy soil from a petroleum-contaminated sandy aquifer as the research object and chose four commonly used ENA materials—calcium peroxide, yeast extract powder, commercial nitrogen fertilizer, and nitrate nutrient solution—to conduct ENA batch experiments. Through metagenomic analysis, we aimed to reveal the evolutionary process of carbon-fixing microorganisms under ENA conditions with common materials, providing theoretical and technical support for the synergistic enhancement of “remediation–carbon sequestration” in petroleum-contaminated sites.

## 2. Materials and Methods

### 2.1. Experimental Design

The polluted medium used in the experiment was sandy aquifer soil from a petroleum-contaminated site in northwestern China. The soil contamination components and inorganic nitrogen elements are detailed in Supplementary Table S1. After sampling, the samples were stored in sealed bags. Before the experiment, the collected samples were air-dried and thoroughly mixed. The processed samples did not contain any gasoline components. The data on the initial strain types and quantities can be found in another research paper [10] on the microbial mechanism of the ENA process. The pollutant selected was commercially available 92# gasoline, and the experimental containers were 40 mL brown screw-capped glass bottles. Since ENA is primarily used in areas with low to moderate concentrations, we selected a concentration of 600 mg/kg gasoline for our experiments.

Based on different ENA materials, a total of five experimental groups were designed: the Natural Attenuation group (NA), where no substances other than deionized water were added; the Calcium Peroxide group (CP), where an oxygen-releasing agent, calcium peroxide (Nong xin ke ji, Jiangmen, China), was added to the experimental system; the Yeast Extract group (YE), where yeast extract (Solarbio^®^ (Cat#Y8020), Beijing, China) was added to the experimental system to supplement additional carbon sources, nitrogen sources, electron acceptors, etc.; the Nitrogen Fertilizer group (NF), where a commercially available compound nitrogen fertilizer (Dugao^®^ Water-soluble fertilizer containing macronutrients (#25-10-20) Shaoyang, China) was added to the experimental system to supplement nitrogen sources, electron acceptors, etc.; and the Self-prepared Nitrogen Solution group (NS), where electron acceptor NO_3_^−^, nitrogen, phosphorus, and trace elements were added to the experimental system (See Supplementary Table S2). To avoid the toxic effects of adding excessive reagents at once on microorganisms, the reagents were added in three installments after each sampling in this experiment.

The concentration of calcium peroxide added each time in the CP group was 1600 mg/kg, which represented the maximum amount that could be added without causing the system’s pH value to exceed 8.5. In the YE group, yeast extract was added at a dose of 100 mg/kg each time, based on referenced dosages. For the NF group, compound nitrogen fertilizer was added at a rate of 464 mg/kg each time, calculated based on its nitrogen content (considered as nitrate) and the stoichiometric relationship with hydrocarbon degradation. In the NS group, the amount of nitrate added was calculated based on the stoichiometric relationship between nitrate reduction and hydrocarbon degradation, while the amounts of other elements added were determined according to the literature [21].

The specific operational procedure was as follows: 10 g of soil and ENA materials were added to a 40 mL brown bottle. The ENA materials containing 2 mL of pure water were transferred to the corresponding bottles, and the bottle caps were tightened. Then, 8 microliters of gasoline were injected into the bottles to achieve a final concentration of 0.8 μL/g, serving as electron donors for the reactions. The brown bottles were incubated in a dark environment at 30 °C. The concentrations of gasoline, NO_3_^−^, NO_2_^−^, NH_4_^+^, and microbial information in the bottles were measured regularly every 6 days. After each sampling, ENA materials containing 0.2 mL of water were injected again into the corresponding remaining bottles.

### 2.2. Physical and Chemical Parameter Analysis

The gasoline concentration was determined using a gas chromatography/mass spectrometry (GC/MS) system (Agilent 7890a-5975c, Agilent Technologies, Santa Clara, CA, USA) equipped with an RTX-624 capillary column, following the United States Environmental Protection Agency (USEPA) Method 524.3 [22]. The concentrations of NO_3_^−^, NO_2_^−^, and NH_4_^+^ during the degradation process were measured using a spectrophotometer (Shimadzu UV2550, Shimadzu, Kyoto, Japan) according to the methods outlined in Soil Agricultural Chemical Analysis [23].

### 2.3. DNA Extraction and Sequencing

Soil samples cultured in parallel were mixed. DNA extraction was performed using approximately 0.56–2.55 g of soil, in accordance with previous studies [24]. After checking the DNA quality, the DNA was fragmented, and paired-end libraries were constructed for shotgun metagenomic sequencing, as described in prior research [4,25]. All sequencing work was carried out on the Illumina HiSeq4000 platform (Illumina Inc., San Diego, CA, USA) at Majorbio Bio-Pharm Technology Co., Ltd. (Shanghai, China).

### 2.4. Data Processing

(1) Concentration–time curves were plotted using Origin software (2019b).
(2) The apparent consumption concentrations of nitrate, nitrite, and ammonium were calculated by subtracting the detected concentrations from the added concentrations. Positive values indicated consumption, while negative values indicated production, i.e., apparent generation.

For microbial sequencing data, emphasis was placed on gene abundance data related to carbon fixation. Based on the definitions from previous research [5,26] and the KEGG metabolic pathway database, there are a total of eight key enzymes across six carbon fixation pathways that are responsible for converting inorganic carbon into organic carbon. In this study, these enzymes were selected as the core research subjects (Table 1). Gene abundance was expressed as rpkm, representing the number of reads containing the gene per million reads. The ratio of the abundance value of each gene collected at different time points to its abundance value at zero time was calculated, and a heatmap was plotted to evaluate the temporal changes in relevant genes [27]. By comparing with the NR database, the overall microbial community structure and the microbial community structure containing certain key enzymes were identified and presented using bar charts [13].

## 3. Results

### 3.1. Degradation of Petroleum Hydrocarbons and Evolutionary Characteristics of Physicochemical Properties

**Figure 1:**
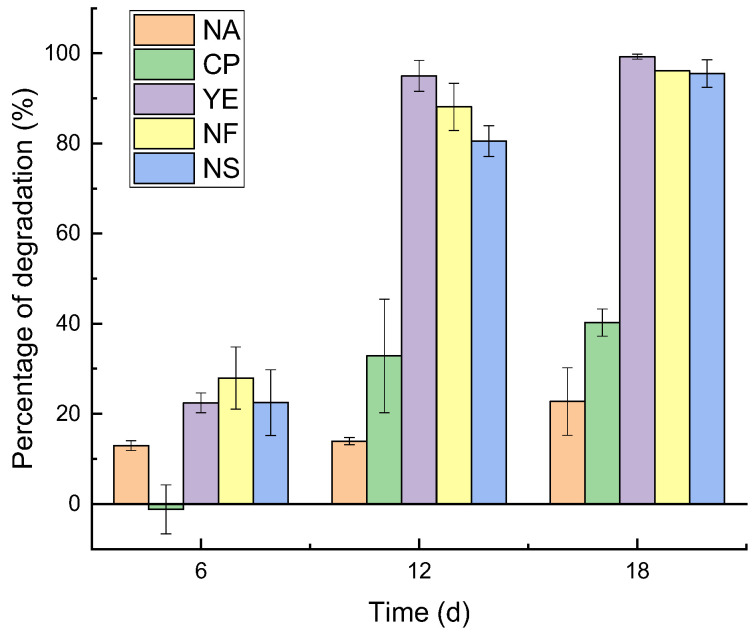
Bar chart showing the degradation extent of pollutants under different treatments, including Natural Attenuation (NA), Calcium Peroxide (CP), Yeast Extract (YE), Nitrogen Fertilizer (NF), and Nitrogen Solution (NS) groups, at various time points.

The degradation of petroleum pollutants under the influence of different ENA materials at various sampling time points is illustrated in Figure 1. In the Natural Attenuation (NA) group, approximately 10% degradation occurred between days 6 and 12 of the experiment, with an average degradation degree of about 20% by day 18. The Calcium Peroxide (CP) group showed no degradation on day 6, with degradation degrees ranging from 20% to 40% on day 12 and reaching around 40% by day 18. The Yeast Extract (YE) group exhibited a degradation degree of approximately 20% on day 6, around 95% on day 12, and over 99% by day 18. The Nitrogen Fertilizer (NF) group had a degradation degree of 20–35% on day 6, over 80% on day 12, and 96% by day 18. The Nitrogen Solution (NS) group showed a degradation degree of 15–30% on day 6, around 80% on day 12, and approximately 95% by day 18. From the final (day 18) results, the YE, NF, and NS groups all achieved favorable degradation effects, with the YE group being the most optimal, reaching over 99%, while the NF and NS groups showed no significant difference, both around 95%. Compared to the NA group, the CP group demonstrated a certain promoting effect on degradation, but the promotion rate was relatively small, only around 20%. From the first monitoring time point, compared to the NA group, the YE, NF, and NS groups all exhibited enhanced degradation effects within 6 days; however, the CP group showed a lower degradation degree than the NA group, indicating a negative effect on degradation, until day 12 when its degradation degree exceeded that of the NA group, demonstrating a certain enhancing effect.

**Figure 2:**
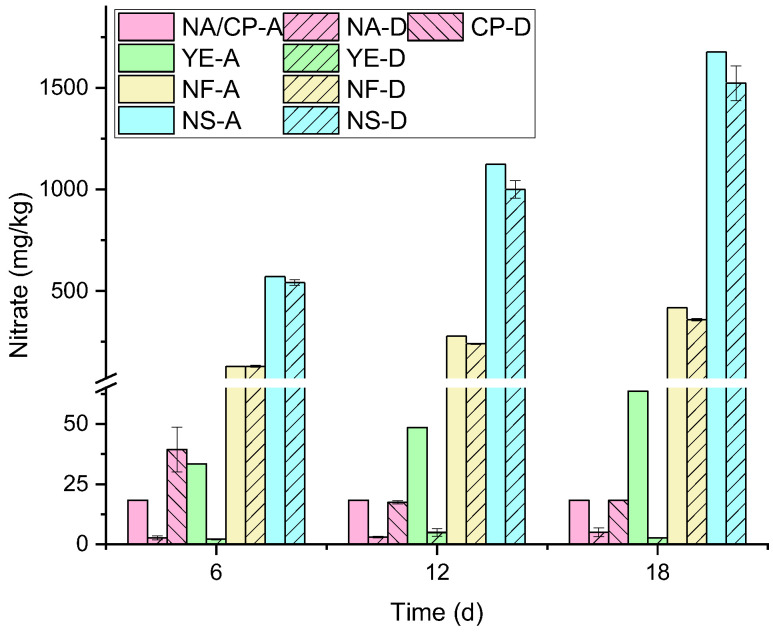
Comparative analysis chart of nitrate content. X-A: The theoretical value of nitrate belonging to Group X at a certain moment under abiotic geochemical conditions; X-D: The measured value of nitrate belonging to Group X. X may represent any of the treatment groups: Natural Attenuation (NA), Calcium Peroxide (CP), Yeast Extract (YE), Nitrogen Fertilizer (NF), or Nitrogen Solution (NS).

As shown in Figure 2, on day 6 of the reaction, there was no significant difference between the measured nitrate values in the NF group and their theoretical total content (*p* < 0.05), while the CP group showed a significant increase compared to its theoretical value (*p* < 0.05). The other three groups all exhibited significant decreases compared to their corresponding theoretical values, with the NA and YE groups showing marked reductions. By day 12 of the reaction, the measured nitrate values in the NF group slightly decreased compared to their theoretical total content (*p* < 0.05), while the CP group showed no significant difference from its theoretical value (*p* < 0.05). The difference between the measured and theoretical values in the NS group increased compared to day 6 (*p* < 0.05), while the difference in the NA group slightly decreased (*p* < 0.05), and the difference in the YE group significantly increased compared to day 6 (*p* < 0.05). By day 18 of the reaction, the difference between the measured and theoretical nitrate values in the NF group increased compared to day 12 (*p* < 0.05), while the CP group showed no significant difference from its theoretical value (*p* < 0.05) (close to the difference on day 12). The difference in the NS group increased compared to day 12 (*p* < 0.05), while the difference in the NA group decreased compared to day 12 (*p* < 0.05), and the difference in the YE group significantly increased compared to day 12 (*p* < 0.05).

The consumption of nitrate typically originates from the nitrate reduction process, in which nitrate serves as an electron acceptor and undergoes redox reactions with electron donors (such as petroleum hydrocarbons) under microbial action, being reduced to nitrite, nitrous oxide, nitrogen gas, or ammonium salts. During this process, electron donors like petroleum hydrocarbons release electrons and undergo oxidative degradation [28].

Overall, the differences between the measured nitrate values and their theoretical total content in the NF and NS groups gradually increased, confirming nitrate consumption. Based on the corresponding relationship between the complete degradation amount of petroleum hydrocarbons and the nitrate consumption amount, the calculated measured nitrate consumption amount was relatively low, possibly related to the incomplete degradation of petroleum hydrocarbons [10,29]. Nitrate, as an electron acceptor, not only participates in the degradation of petroleum hydrocarbons but can also utilize CO_2_ for carbon fixation. Compared to the NF group, the NS group had a higher nitrate consumption amount, while there was no significant difference in petroleum hydrocarbon degradation amounts, suggesting that a larger proportion of nitrate in the NS group contributed to other processes such as carbon fixation. The measured nitrate concentrations in the YE group showed no significant differences on days 6, 12, and 18, but the theoretical total amount continued to increase, indicating a shortage of nitrate supply. Similarly, carbon fixation in this group may have consumed a relatively large amount of nitrate. The measured nitrate values in the CP group significantly increased compared to their theoretical values on day 6, suggesting that the oxygen released from calcium peroxide triggered nitrification in the system, converting nitrogen in the system into NO_3_^−^. Subsequently, in the aerobic environment, microorganisms utilized oxygen and NO_3_^−^ as the main electron acceptors to degrade petroleum hydrocarbons, leading to decreases in both NO_3_^−^ and petroleum hydrocarbon concentrations, resulting in similar measured and theoretical NO_3_^−^ values on days 12 and 18 [30]. Similarly, nitrate and oxygen in this group participated in the carbon fixation process as electron acceptors.

### 3.2. Evolution of Functional Abundance of Carbon-Fixing Microorganisms

The changes in the abundance of key carbon-fixing enzyme-encoding genes involved in various carbon fixation pathways are statistically presented in Table 2.

As shown in the table, there was no significant change in the abundance of carbon-fixing genes in the NA group within 6 days. On day 12, the abundance of genes encoding the 1.2.7.3 enzyme involved in the Reductive tricarboxylic acid (rTCA) pathway, the 1.2.7.1 enzyme involved in both the rTCA and Dicarboxylate/4-hydroxybutyrate (DH) cycles, and the 6.4.1.3 enzyme involved in the 3-hydroxypropionate cycle (HP) or 3-hydroxypropionate/4-hydroxybutyrate (HH) pathway significantly decreased (decrease degree > 20%), while only the abundance of the gene encoding the 4.1.1.31 enzyme involved in both the rTCA and DH cycles increased by more than 20%. In the CP group on day 6, the abundance of genes encoding the 1.1.1.42 enzyme involved in the rTCA pathway and the 4.1.1.31 enzyme involved in both the rTCA and DH cycles significantly decreased (by more than 20%), while the abundance of other genes increased. On day 12, the abundance of genes encoding the 4.1.1.39 enzyme involved in the Calvin–Benson–Bassham (CBB) cycle and the 1.1.1.42 enzyme involved in the rTCA pathway decreased, while the abundance of other genes increased to varying degrees. The YE, NF, and NS groups exhibited similar abundance change characteristics in carbon-fixing enzyme-encoding genes, specifically showing varying degrees of decrease in the abundance of genes encoding the 4.1.1.39 enzyme involved in the CBB cycle, the 1.2.7.3 enzyme involved in the rTCA pathway, the 1.2.7.1 enzyme involved in both the rTCA and DH cycles, and the 6.4.1.3 enzyme involved in the HP or HH pathway (except for the 1.2.7.1 enzyme in the YE group on day 18), while the abundance of genes encoding the 1.1.1.42 enzyme involved in the rTCA pathway and the 4.1.1.31 enzyme involved in both the rTCA and DH cycles increased to varying degrees. Additionally, since the initial abundance of the gene encoding the 1.2.7.4 enzyme involved in the Wood–Ljungdahl (WL) cycle was 0, this gene was not included in the statistics in Table 2. The results showed that this gene was only present in the YE group, with abundances of 4, 5, and 20 rpkm, showing a gradual increasing trend. From the perspective of total carbon-fixing gene abundance, except for the CP group, which showed an increase in carbon-fixing gene abundance, the abundance in other treatments decreased to varying degrees, but the decrease was controlled within 20%, with the NA and YE groups showing decreases within 10% and the NF and NS groups within 20%.

### 3.3. Evolution of Carbon-Fixing Microbial Species

**Figure 3:**
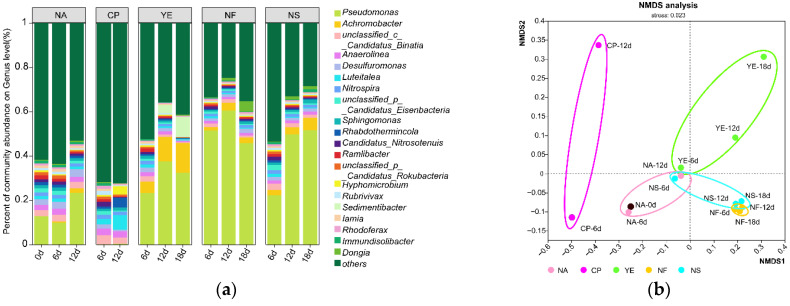
Microbial community analysis under different treatments: (**a**) Genus-level bar chart showing taxonomic annotation of carbon-fixation gene-containing microorganisms; (**b**) Non-metric multidimensional scaling (NMDS) ordination plot. Treatments include: Natural Attenuation (NA), Calcium Peroxide (CP), Yeast Extract (YE), Nitrogen Fertilizer (NF), and Nitrogen Solution (NS).

From the carbon-fixing microbial community structure diagram (Figure 3a), it can be seen that *Pseudomonas* was the most dominant species in the NA, YE, NF, and NS groups, and its dominance gradually increased over time (except on day 18), while it was almost absent in the CP group.

The abundance of *Achromobacter* was relatively low at all time points in the CP group, as well as on day 0 and day 6 in the NA group. In the other groups, its abundance ranked second only to that of *Pseudomonas*, making it a dominant species. Its abundance increased over time in the NA, YE, NF, and NS groups, reaching 15% in the YE group, significantly higher than in other groups. Other genera such as *unclassified_c__Candidatus_Binatia*, *Anaerolinea, and Desulfuromonas* had similar initial abundances (2–3%). After ENA treatment, the abundance differences in these genera increased in the CP group, while their abundances remained relatively close but decreased (around 1%) in the YE, NF, and NS groups. Comparing the annotation results of microorganisms containing carbon-fixing genes with those of all microorganisms (Supplementary Figure S1), it was found that they had similar population structures: *Pseudomonas* and *Achromobacter* were both dominant species and showed similar changing trends over time. At the species level (Supplementary Figure S1), there were multiple dominant species under the *Pseudomonas* genus, including *Pseudomonas aromaticivorans*, *Pseudomonas sagittaria*, *Pseudomonas linyingensis*, *Pseudomonas frederiksbergensis*, etc.

The Non-metric multidimensional scaling (NMDS) analysis diagram (Figure 3b) more clearly shows the similarity of community structures among samples. Overall, the CP group was relatively distinct from other groups. Centered around the initial point (NA-0d), the CP group mainly evolved along the negative direction of the NMDS1 axis, while other groups, including the NA group, evolved towards the positive direction of the NMDS1 axis. From the perspective of the NMDS2 axis, the NA, NF, and NS groups showed little change in the NMDS2 axis direction (within a range of 0.1), while the CP and YE groups showed larger changes in the NMDS2 axis direction (with a span of over 0.4 compared to the initial point). Combining the analysis results of the community composition bar chart (Figure 3a), it can be seen that ENA materials altered the microbial community structure. The materials in the YE, NF, and NS groups increased the abundance of the original dominant microorganisms, while the materials in the CP group led to the disappearance of dominant genera (such as *Pseudomonas*) and deviated from the original community structure.

## 4. Discussion

### 4.1. Petroleum Hydrocarbon Degradation Process

This study primarily simulates the anaerobic environment of an aquifer, where insufficient electron acceptors and nitrogen sources are the main limiting factors for pollutant degradation [31,32,33]. Monitoring of nitrogen sources and nitrate concentrations revealed that the nitrogen in the NA group was rapidly depleted, leading to a near halt in pollutant degradation due to a lack of nitrogen and electron acceptors. Therefore, supplementing nitrogen sources and electron acceptors is the main direction for ENA [34,35]. Materials in the YE, NF, and NS groups can simultaneously provide nitrogen sources (ammonium or nitrate) and electron acceptors (nitrate) for microbial growth, while the CP group can only provide electron acceptors (oxygen). Generally, oxygen is preferentially utilized as an electron acceptor over nitrate, but the CP group lacks nitrogen sources, limiting growth [36]. Moreover, the addition of calcium peroxide altered the soil pH, potentially causing changes in the microbial community structure (as confirmed by microbial community structure analysis), leading to the original petroleum hydrocarbon-degrading microorganisms becoming unable to adapt to the new environment and ceasing metabolism. This explains why no degradation occurred in the CP group on day 6 [37]. Subsequently, the microorganisms gradually adapted to the environment and began degradation, but the degradation efficiency was only about 40% after 18 days, likely due to a lack of nitrogen sources. Another possible reason is that the sampling site was heavily contaminated, leading to near-complete depletion of oxygen. The indigenous microorganisms had adapted to the anaerobic environment, while CP treatment shifted the original anaerobic conditions to aerobic conditions, thereby inhibiting the anaerobic microbiota. Consequently, CP suppressed a significant proportion of dominant anaerobic microorganisms, including those responsible for degradation and carbon fixation.

### 4.2. Carbon Fixation Gene Abundance

It has been reported that the CBB cycle primarily occurs in microbial chemolithoautotrophic metabolism under aerobic and microaerobic conditions [38,39]. However, the experimental system in this study was sealed, and for treatments other than the CP group, microorganisms preferentially utilized the limited O_2_ in the experimental bottles as electron acceptors before entering anaerobic conditions. The growth of carbon-fixing microorganisms involved in the CBB cycle was inhibited, leading to a gradual decrease in their abundance, even by over 90% (YE group on day 18). For the CP group, the slow release of oxygen from added calcium peroxide may have promoted the growth of microorganisms involved in the CBB cycle, resulting in an increase in abundance on day 6. However, a severe decrease in abundance was observed on day 12, possibly due to the continuous addition of calcium peroxide affecting the environmental conditions for the CBB cycle, such as high pH, which may cause some microorganisms to become unable to adapt [40].

Among the carbon fixation enzyme-encoding genes involved in the rTCA and DH cycles, the abundances of isocitrate dehydrogenase (EC: 1.1.1.42) and phosphoenolpyruvate carboxylase (EC. 4.1.1.31) increased after ENA in the NA, CP, YE, NF, and NS groups, while the abundances of pyruvate synthase (EC: 1.2.7.1) and 2-oxoglutarate synthase (EC: 1.2.7.3) decreased. A possible reason is that in the rTCA and DC/4HB cycle pathways, EC: 1.2.7.1 and EC. 1.2.7.3 are located 1–2 steps before EC. 4.1.1.31 and EC: 1.1.1.42, respectively. It is speculated that there were already large amounts of the synthesis products of EC: 1.2.7.1 and EC. 1.2.7.3, pyruvate and 2-oxoglutarate, which are common organic metabolites, in the microbial system, eliminating the need for further synthesis [41,42]. The opposite trend was observed in the CP group because the carbon fixation enzymes involved in the rTCA and DC/4HB cycles are mostly reversible reaction enzymes that participate in the TCA cycle for organic matter degradation under aerobic conditions and the rTCA cycle for carbon fixation under anaerobic conditions [43]. It is speculated that in the CP system, these enzymes primarily participate in the TCA cycle for organic matter degradation, resulting in an opposite trend in abundance compared to other groups.

Among the carbon fixation enzyme-encoding genes involved in the HP or HH cycles, the abundance of propionyl-CoA carboxylase (EC: 6.4.1.3) first increased and then decreased after ENA in the NA group. In the CP group, its abundance continuously increased after ENA. In the YE, NF, and NS groups, its abundance continuously decreased after ENA. A possible reason is that the HP or HH cycle pathway is an aerobic carbon fixation pathway. Therefore, in the NA group, aerobic metabolism occurred first and then gradually terminated as oxygen was consumed. In the CP group, continuous oxygen release allowed for sustained metabolism of carbon-fixing microorganisms, and the easily produce CO_2_ can later be used for carbon-fixing microorganisms. In the YE, NF, and NS groups, rapid oxygen consumption due to rich nutrients led to metabolic termination [26]. The abundance of acetyl-CoA carboxylase (EC: 6.4.1.2) showed little change in all groups, suggesting that this enzyme is not very sensitive to oxygen.

The YE group demonstrated optimal performance both in hydrocarbon degradation efficiency and carbon fixation gene levels, which can be attributed to the fact that yeast extract not only provides essential nitrogen and electron acceptors, but also supplies multiple trace elements required for microbial growth, as detailed in our previous study [10].

### 4.3. Species with Carbon Fixation Genes Annotated

In this experimental system, the most abundant microorganisms with carbon fixation genes were *Pseudomonas*. Carbon-fixing species within this genus, including *Pseudomonas fluorescens* and *Pseudomonas saccharophila*, have been identified, while *Pseudomonas aromaticivorans*, *Pseudomonas sagittaria*, *Pseudomonas linyingensis*, and *Pseudomonas frederiksbergensis* identified in this study have not been reported to fix carbon [44,45]. However, according to the literature, *Pseudomonas aromaticivorans*, *Pseudomonas sagittaria*, *Pseudomonas linyingensis*, and *Pseudomonas frederiksbergensis* can degrade petroleum hydrocarbons or their derivatives [46,47,48,49]. In this study, the *Pseudomonas* genus was the most dominant among all microorganisms and also the most dominant nitrate-reducing petroleum hydrocarbon-degrading microorganism in the experimental system, indicating its potential for nitrate-reducing organic matter degradation [10].

*Achromobacter* was the second most abundant microorganism with carbon fixation genes in this experimental system. The identified species in this study did not match any similar species in existing databases, and no relevant reports have been found. However, the literature indicates that this genus has the ability to perform autotrophic carbon fixation from carbon dioxide [14,50], and related studies have confirmed its carbon dioxide fixation function using sodium thiosulfate as an energy source [51]. Previous studies have shown that this genus is also a dominant nitrate-reducing petroleum hydrocarbon-degrading microorganism in the experimental system [10].

*Candidatus* was the third most abundant microorganism with carbon fixation genes in this experimental system. *Candidatus Binatia*, a species within this genus, was found to possess carbon fixation functional enzymes, but its carbon fixation function was not expressed, and it only exhibited carbon monoxide oxidation function under aerobic conditions. Therefore, this species does not belong to carbon-fixing species, explaining the increase in gene abundance in the CP group in Table 2 [52,53]. Previous studies have shown that this genus is also a nitrate-reducing petroleum hydrocarbon-degrading microorganism in the experimental system [10].

*Anaerolinea* was the fourth most abundant microorganism with carbon fixation genes in this experimental system. The identified species within this genus did not match any similar species in existing databases, but other species within the genus, *Anaerolinea Chloroflexi*, have been reported in the literature, indicating that this carbon-fixing species fixes carbon dioxide through heterotrophic metabolism [54,55]. Previous studies have shown that this genus is also a nitrate-reducing petroleum hydrocarbon-degrading microorganism in the experimental system [10].

This study indicates that a considerable number of petroleum hydrocarbon-degrading microorganisms are also carbon-fixing microorganisms, consistent with our previous findings [2,3,5]. Therefore, petroleum hydrocarbon-degrading bacteria stimulated by ENA materials are highly likely to also possess carbon fixation functions.

### 4.4. Prospects

This study identified carbon-fixing microorganisms and their evolutionary processes during the stimulation of petroleum hydrocarbon degradation by different ENA materials from a metagenomic perspective. It recognized that a large number of carbon-fixing microorganisms grow and metabolize during the ENA process, enriching the understanding of carbon cycle microorganisms in petroleum-contaminated sites and providing a theoretical basis for green and low-carbon management of such sites. In the study, we hypothesize that reduced substances generated during petroleum hydrocarbon degradation—such as hydrogen gas and nitrous oxide—may serve as electron donors for carbon fixation, while oxidized substances like nitrate act as electron acceptors. However, this study has not evaluated the stoichiometric relationship between carbon sources and carbon sinks in the experimental system and cannot determine whether these carbon-fixing microorganisms truly perform carbon fixation, i.e., whether they possess carbon fixation potential. This is also a problem that needs to be addressed in future work. On the other hand, this study only focused on individual enhancement materials, and many other enhancement materials remain to be explored; differences may also exist in different environments. However, overall, this study provides a low-carbon perspective to consider for remediation.

## 5. Conclusions

This study compared the effects of four enhanced natural attenuation materials—calcium peroxide (CP), yeast extract (YE), commercially available compound nitrogen fertilizer (NF), and a self-prepared nitrogen-containing solution (NS)—on the succession of microbial communities in a petroleum-contaminated aquifer. It revealed the response mechanism of carbon-fixing microorganisms and their synergistic relationship with hydrocarbon degradation functions.

Nitrate-based materials (YE/NF/NS) significantly increased the petroleum hydrocarbon degradation rate (>95%) but suppressed certain carbon fixation genes (such as those involved in the CBB cycle). Due to the lack of a nitrogen source and pH disturbances, calcium peroxide (CP) only achieved 40% degradation but promoted the expression of carbon fixation genes in the rTCA pathway (such as 1.1.1.42 and 4.1.1.31). Dominant bacterial genera (*Pseudomonas*, *Achromobacter*) exhibited both hydrocarbon degradation and carbon fixation functions. The use of nitrate-based materials for enhanced natural attenuation enriched these facultative bacteria, which degraded pollutants while maintaining carbon-fixing biomass. Yeast extract significantly increased the gene abundance in the Wood–Ljungdahl (WL) pathway, revealing that organic carbon sources can activate anaerobic carbon fixation pathways. The type of electron acceptor drove the differentiation of carbon fixation pathways—nitrate-dominated systems promoted the rTCA and WL pathways, while oxygen-dominated systems activated the CBB and rTCA pathways. Despite a slight decrease in the total abundance of carbon fixation genes (<20%), the upregulation of key genes indicated that microorganisms could maintain their carbon fixation potential by switching metabolic pathways, confirming that ENA does not weaken the carbon fixation process.

## Figures and Tables

**Table 1:** Key enzymes in carbon fixation pathways.

Cycle (s) Involved	Enzyme Description	E.C.
Calvin–Benson–Bassham (CBB)	Ribulose-bisphosphate carboxylase	4.1.1.39
Reductive tricarboxylic acid (rTCA)	Isocitrate dehydrogenase (NADP^+^)	1.1.1.42
	2-oxoglutarate synthase	1.2.7.3
Reductive tricarboxylic acid (rTCA), Dicarboxylate/4-hydroxybutyrate (DH)	Pyruvate synthase	1.2.7.1
	Phosphoenolpyruvate carboxylase	4.1.1.31
Wood–Ljungdahl (WL)	Anaerobic carbon-monoxide dehydrogenase	1.2.7.4
3-hydroxypropionate cycle (HP), 3-hydroxypropionate/4-hydroxybutyrate (HH)	Propionyl-CoA carboxylase	6.4.1.3
	Acetyl-CoA carboxylase	6.4.1.2

**Table 2:** Variations in the abundance of genes related to carbon fixation *.

Pathways	Enzyme	NA		CP		YE			NF			NS		
		6d	12d	6d	12d	6d	12d	18d	6d	12d	18d	6d	12d	18d
CBB	4.1.1.39	0.96	0.88	1.30	0.36	0.67	0.31	0.09	0.26	0.19	0.38	0.54	0.38	0.30
rTCA	1.1.1.42	1.00	1.15	0.74	0.82	1.22	1.29	1.31	1.21	1.32	1.07	1.22	1.25	1.22
	1.2.7.3	1.01	0.64	1.43	1.80	0.66	0.51	0.70	0.33	0.22	0.32	0.75	0.32	0.24
rTCA/DH	1.2.7.1	0.96	0.54	1.41	1.09	0.67	0.68	1.47	0.33	0.23	0.27	0.73	0.30	0.21
	4.1.1.31	1.01	1.21	0.69	1.29	1.17	1.17	0.98	1.53	1.58	1.17	1.16	1.41	1.20
HP/HH	6.4.1.3	1.11	0.80	1.34	2.01	0.70	0.54	0.36	0.86	0.77	0.99	0.83	0.80	0.80
	6.4.1.2	0.99	0.96	1.07	1.04	1.09	1.15	1.12	0.98	0.99	1.01	1.00	0.98	0.97
Total		1.01	0.90	1.11	1.28	0.95	0.93	0.99	0.87	0.86	0.84	0.95	0.86	0.80
								Color scale		0.00	0.50	1.00	1.50	2.00

* The colors depicted in the table correspond to the ratios of gene abundance in the sample compared to the abundance at the initial (0 time point) in different treatments, including Natural Attenuation (NA), Calcium Peroxide (CP), Yeast Extract (YE), Nitrogen Fertilizer (NF), and Nitrogen Solution (NS) groups. CBB, rTCA, DH, HP, and HH are acronyms representing the Calvin–Benson–Bassham cycle, the Reductive Tricarboxylic Acid cycle, the Dicarboxylate/4-Hydroxybutyrate cycle, the 3-Hydroxypropionate cycle, and the 3-Hydroxypropionate/4-Hydroxybutyrate cycle, respectively.

## Data Availability

The data presented in this study are available on request from the corresponding author, data are not publicly available due to privacy.

## Supplementary Materials

The following supporting information can be downloaded at: https://www.mdpi.com/article/10.3390/microorganisms13092205/s1, Figure S1: Bar chart of carbon-fixing microorganisms at the species level; Table S1: The soil contamination components and inorganic nitrogen elements; Table S2: The formula for self-compounding reagent.
