# Supplementary material for: Synergistic Potential of Contamination Remediation and Carbon Fixation: Functional Resilience of Carbon Fixation in Petroleum Hydrocarbon-Degrading Microbial Communities Under Enhanced Natural Attenuation
Source: Microorganisms. 2025 Sep 20;13(9):2205. doi: 10.3390/microorganisms13092205 (PMC12472652; doi:10.3390/microorganisms13092205)
Supplement: Supplementary file 1 [file microorganisms-13-02205-s001.zip › microorganisms-3850866-supplementary.pdf]

Supplementary Material

1 Supplementary Figures and Tables

1.1 Supplementary Figures

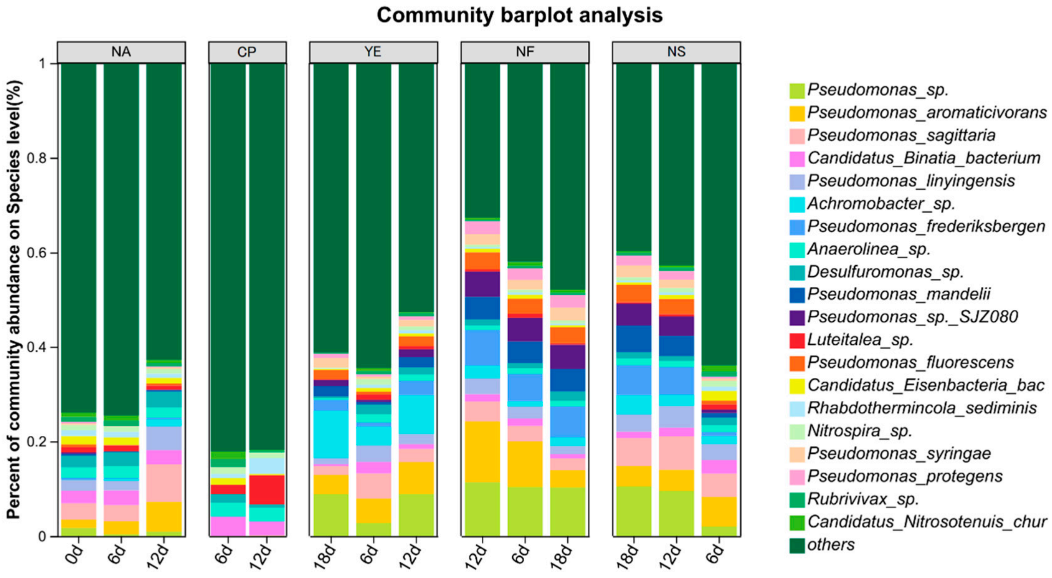

Figure S1 Bar chart of carbon-fixing microorganisms at the species level.

1.2 Supplementary Tables

Table S1 The soil contamination components and inorganic nitrogen elements

Sampling Depth (m)	C6-C9 (mg/kg)	Ammonium (mg/kg)	Nitrite (mg/kg)	Nitrate (mg/kg)	VOC* (mg/kg)
3-4.5	6664	0.725	0.075	18.3	26.1

* The specific components of VOC are as follows (μg/kg):

**Component**	**Concentration (μg/L)**	**Component**	**Concentration (μg/L)**	**Component**	**Concentration (μg/L)**	**Component**	**Concentration (μg/L)**
Vinyl Chloride	0.04 ND#	Benzene	737	Chlorobenzene	1.20 ND	4-Chlorotoluene	1.30 ND
1,1-Dichloroethylene	1.00 ND	Trichloroethylene	1.20 ND	1,1,1,2-Tetrachloroethylene	1.20 ND	1,3,5-Trimethylbenzene	1710
Dichloromethane	1.00 ND	Epichlorohydrin	2.30 ND	cis-1,2-Dichloroethylene	0.80 ND	tert-Butylbenzene	1.20 ND
trans-1,2-Dichloroethylene	1.10 ND	1,2-Dichloropropane	1.10 ND	Ethylbenzene	1285	1,2,4-Trimethylbenzene	2824
1,1-Dichloroethane	0.80 ND	Dibromomethane	1.20 ND	m,p-Xylene	10583	sec-Butylbenzene	227
Chloroprene	1.10 ND	Bromodichloromethane	1.10 ND	Styrene	263	1,3-Dichlorobenzene	1.50 ND
cis-1,2-Dichloroethylene	0.80 ND	Chloroprene	1.10 ND	o-Xylene	2431	4-Isopropyltoluene	156
2,2-Dichloropropane	1.30 ND	cis-1,3-Dichloropropene	1.20 ND	Bromoform	1.50 ND	1,4-Dichlorobenzene	1.50 ND

Chloroform	1.10 ND	trans-1,3-Dichloropropene	1.20 ND	1,1,2,2-Tetrachloroethane	568	1,2-Dichlorobenzene	1.70 ND
Bromochloromethane	1.40 ND	Toluene	1403	Isopropylbenzene (Cumene)	524	Chloroform	1.10 ND
1,1-Dichloroethane	0.80 ND	1,1,2-Trichloroethane	2313	1,2,3-Trichloropropane	1.20 ND	n-Butylbenzene	359
1,1,1-Trichloroethane	1.30 ND	1,3-Dichloropropane	1.10 ND	Bromobenzene	1.30 ND	1,2-Dibromo-3-chloropropane	1.90 ND
1,2-Dichloroethane	1.30 ND	Dibromochloromethane	1.10 ND	n-Propylbenzene	435	1,2,4-Trichlorobenzene	0.30 ND
1,1-Dichloropropene	1.20 ND	Tetrachloroethylene	1.40 ND	2,2-Dichloropropane	1.30 ND	Naphthalene	141
Carbon Tetrachloride	1.30 ND	1,2-Dibromoethane	1.10 ND	2-Chlorotoluene	142	Hexachlorobutadiene	0.40 ND

ND indicates "Not Detected".The value preceding it represents the minimum detection limit. Values without the "ND" label are the actually detected concentrations.

Table S2 The formula for self-compounding reagent.

Formula		The trace element solution	
Chemical component	Concentration (g/L)	Chemical component	Concentration (g/L)
KNO_3	45.06	$FeSO_4 \cdot 4H_2O$	1.8
NH_4Cl	2.39	$CoCl_2 \cdot 6H_2O$	0.25
KH_2PO_4	0.61	$CuCl_2 \cdot 2H_2O$	0.01
Na_2SO_4	0.63	$NiCl \cdot 6H_2O$	0.01
Trace element solution	10	$MnCl_2 \cdot 4H_2O$	0.7
		$ZnCl_2$	0.1
		H_3BO_3	0.5
		$(NH_4)_6Mo_7O_{24} \cdot 4H_2O$	0.1
